# Acute and Chronic Albuminuria, or Bright’s Diseases of the Kidney

**Published:** 1874-03

**Authors:** J. B. Baird


ATLANTA

MEDICAL AND SURGICAL JOURNAL.

VOL. XI.] MARCH—1874. [NO. 12.

### Original Communications.

### ACUTE AND CHRONIC ALBUMINURIA;

OR,

### BRIGHT'S DISEASES OF THE KIDNEY.

AN ESSAY READ BEFORE THE ATLANTA ACADEMY OF MEDICINE,

BY J. B. BAIRD, M.D.*

In acute Bright's disease or acute desquamative nephritis, acute tubal nephritis, acute albuminuria, or acute inflammatory dropsy, though not devoid of danger, the great majority of cases recover. The chronic affection involving irremediable structural changes, sooner or later ends fatally. The former has long been familiar to practitioners as a sequel apt to follow scarlet fever, but these diseases were not understood by the profession prior to the writings of Richard Bright, in 1827, to whom, and to subsequent observers, we are indebted for our knowledge of these most important diseases.

In the acute disease, the kidneys are increased in volume and weight, sometimes exceeding eight ounces, the normal weight being from three to five ounces. The renal substance is everywhere engorged with blood—but in some cases the cortical substance is anæmic, though generally it presents engorged and

* This Essay, read before the Academy of Medicine, was not designed for publication, hence extracts, often in the words of the authors, from the works of Flint, Clark, Dalton, Johnson, and others, are not accredited to their source, nor even marked with quotation points.

exsanguined patches. The microscope shows tubes filled with epithelial cells, and a substance supposed to be exuded fibrin. It will be observed that in this affection there is no structural change; the anatomical characters being due to an accumulation of epitheleum, and perhaps fibrin in the tubes, and more or less congestion. Both kidneys are affected in the same manner and degree.

In regard to the chronic form of the disease, there is a difference of opinion among pathologists; some regarding the morbid conditions as different stages of the same disease; others holding to the opinion that they are essentially different affections. Evidence seems undoubtedly to preponderate in favor of the latter opinion, though as it is frequently impossible to discriminate between them at the bedside, it is convenient to consider them under one head. The chronic affection, at all events is not rare, and it is important as standing in a causative relation to dropsy and enæmia, and its liability to be associated with other affections. The morbid appearances are various, and pathologists have been led by the gross appearances to divide the disease into several forms—some make eight, others four, five, three and six. Flint describes four forms: The large white kidney; the hard contracted kidney; the fatty kidney, and lardaceous, amyloid or waxy kidney. The first, as its name imports, is increased in size and weight. It may equal or exceed twelve ounces—the normal weight being from three to five ounces; the color is white or whiteish. It is the form presented when the chronic has been preceded by the acute affection. The enlargement is in the cortical portion, which is—as are also the cones sometimes anæmic. The microscope shows an accumulation within the tubes of disintegrated epithelium and granular matter. The hard, contracted kidney presents a striking contrast to the former. It is shrunken in size, and sometimes weighs only one ounce and a half. It is abnormally dense and resisting; the surface may be smooth or present a lobulated "hobnailed" appearance. The microscope shows an abundance of epithelial tissue and granules in more or less of the uriniferous tubes. Fibrous tissue appears to be more abundant than in the healthy kidney; some pathologists claim an increase of substance. There is an obvious resemblance to cirrhosis of the liver. It is sometimes called granular, atrophied or fibrous kidney.

Fatty kidney: In this affection the organ is more or less enlarged. It is of a yellowish-fawn color, and a greasy feel. Oil drops or granules of fat are present in the acute disease, and to a certain extent, in the large white kidney. In the so-called fatty kidney they are in notable excess. This disease bears a striking resemblance to fatty liver, and the two are apt to be associated.

Lardaceous, waxy or amyloid kidney is comparatively a rare form of the disease. The size of the kidney is not necessarily increased. The tissues are infiltrated by a morbid material, the nature of which has not as yet been satisfactorily ascertained; but when a section of the organ is made, an appearance resembling wax is presented.

The point of departure for the morbid process in the large white fatty kidney is the secreting tubes. In the hard contracted kidney, it is probably in the intertubular substance. In the amyloid kidney, the deposit first commences in and around the vessels, and afterwards in the surrounding tissues. These morbid conditions are symmetrical, both kidneys being affected in the same manner and degree. The fact of its being a bilateral disease goes to sustain the doctrine of dependence on the blood. The different forms of chronic, as well as the acute affection, agree as regards the pathological effects liable to ensue. They can be resolved into two classes: First, interference with the secretory functions of the kidneys; especially as regards the elimination of urea. Second, interference with the circulation in the kidneys. The first occurs in two ways, *i.e.*, either by obstruction of the convoluted tubes by disintegrated epithelium, granular matter, fibrinous exudation and fat, or by destruction of more or less of the secreting structure. The circulation is interfered with also in two ways: not only by pressure of the morbid products on the veins, but in obedience to the law, "whenever, in any organ, the function of that organ is interfered with, the interference extends to the circulation."

Going further, two classes of ulterior effects are liable to occur—uræmia and albuminuria. Instead of water holding in solution the solid constituents of the urine being eliminated, owing to the interference with the circulation, albumen is exuded; the density of the blood plasma is diminished from 1,030 to, sometimes, 1,010; the accumulation of water in the blood, which should be eliminated by the kidneys, and the loss of albumen, which impedes the circulation through the capillaries by diminishing the viscosity of the blood, produces general dropsy.

Dr. Geo. Johnson, in the *British Medical Journal*, for January, 1869, claims that the coats of the small arteries in the various parts of the body are thickened and hypertrophied, in Bright's diseases, and that this condition gives rise to obstruction, which leads to cardiac hypertrophy, general dropsy, etc. He discards the opinion that the altered blood will not pass readily through healthy capillaries. In the large, white kidney, and in the acute affection, the circulation through these organs is greatly impeded, hence the large amount of dropsical effusion usually present in these forms of the disease. In the hard, contracted kidney, the interference is chiefly with the secretory function. Albuminuria occurs in the course of many diseases, and is not always indicative of Bright's disease; but in other affections it is transitory and usually small in quantity. In Bright's disease, though not always abundant, or indeed, invariably present, it is persistent. In testing the urine for albumen by the usual methods, by heat and nitric acid, certain sources of fallacy should be remembered, namely, heating the urine, if it be alkaline, sometimes causes a deposit of phosphates which resembles coagulated albumen. If the urine be alkaline heat, carried to the boiling point, will not always coagulate the albumen. Adding nitric acid to a fresh specimen of urine sometimes leads to the disengagement of uric acid in such quantities as to render the urine opaque, as if albumen were present. To avoid error both heat and nitric acid should be used. As a rule the albumen is abundant in proportion to the amount of the dropsy.

Not very infrequently, in the hard, contracted kidney, the urine is not albuminous, and the diagnosis in these cases must rest on evidences furnished by other symptoms, and the presence of casts in the sediment of the urine. Occasionally the amount of urine is increased, especially in connection with the contracted kidney; though in the great majority of cases it is diminished, and falls below the normal standard, which may be stated to be from thirty to sixty ounces daily. As a rule the specific gravity is diminished, which, taken into connection with the lessened amount of urine discharged, indicates a deficiency of the solid constituents, the most important of which is urea. By noting the quantity of urine passed in twenty-four hours, and taking the specific gravity, the amount of urea discharged may be determined with sufficient accuracy for ordinary purposes. The amount may be determined with greater precision by volumetric analysis. The amount in the normal urine varies from 350 to 600

grains daily, and is influenced largely by muscular exercise and the amount of nitrogenized food ingested. Urine deficient in urea decomposes slowly, and does not possess the strong ammoniacal odor of putrescent healthy urine. Urea may be deficient and yet the urine contain no albumen. This is especially observed in connection with the hard contracted kidney.

In the acute affection, subcutaneous œdema is usually the first symptom pointing to the disease. It is generally first observed in the face below the eyes, and then in the lower extremities—sometimes it occurs first in the lower extremities. Coincident with this there is more or less febrile movement, anorexia, thirst, lumbar pains and vomiting. The skin is usually dry; the countenance is pallid; anasarca sometimes, though not invariably, great. Hydro-peritoneum and hydro-thorax occur in a certain proportion of cases. Effusion may take place into the pleural cavity to such an extent as to occasion great suffering from dyspnœa. The urine is usually scanty and sometimes absent. The specific gravity rarely exceeds 1,020, and is sometimes less. Diminution of the specific gravity of the urine, the quantity not being increased, denotes deficiency of urea. Heat and nitric acid show the presence of albumen, frequently in great abundance.

The most important microscopical characters are epithelial casts. These are regarded as especially characteristic of this affection, and consist of desquamated epithelium of the convoluted tubes. Other casts are sometimes observed, thought to be coagulated fibrin from the convoluted tubes. They are called hyaline or waxy casts, and measure from $\frac{1}{500}$ to $\frac{1}{1000}$ of an inch in diameter. Their size is important; the larger being nearly as large as the tubes themselves, indicate that the epithelial lining has been lost. Symptoms of uræmia, namely, coma and convulsions, occur in a certatin proportion of cases, due to an insufficient elimination of urea. Frerichs, a German physician, several years ago, in an able monograph on this subject, contended that the symptoms commonly referred to urea were not due to the presence of that agent, but to carbonate of ammonia into which the urea was converted in the veins. He produced similar symptoms in animals by injecting a solution of carbonate of ammonia into their veins. It has been claimed by some that urea is "probably not more poisonous than nitrate of potash." These views are scarcely tenable at the present day. Dalton says that "Urea has no tendency to spontaneous decomposition, and may be kept, when perfectly pure, in a dry state or dissolved in water, for an indefinite length of time. If the watery solution be boiled, however, the urea is converted, during the process of ebullition, into carbonate of ammonia. One equivalent of urea unites with two equivalents of water, and becomes transformed into two equivalents of carbonate of ammonia, as follows:

$$\begin{array}{r|cccc} & C_2 & H_4 & N_2 & O_2 \\ & & H_2 & & O_2 \\ \hline 2) & C_2 & H_6 & N_2 & O_4 \\ \hline & C & H_3 & N & O_2 = NH_3\ ,\ CO_2 \end{array}$$

Various impurities, also, by acting as catalytic agents, will produce the same change, if water be present. Animal substances, in a state of commencing decomposition, are particularly liable to act in this way. In order that the conversion of the urea be thus effected, it is necessary that the temperature of the mixture be not far from 70° to 100° F. Vomiting and purging may occur irrespective of cerebral symptoms, being probably due to an effort of nature to eliminate urea through the gastro-intestinal mucous membrane.

The chronic affection sometimes follows the acute. It is thought by some pathologists that this statement applies solely to the large white kidney. As a rule the affection is sub-acute from the first; as in the acute disease dropsy, in a large proportion of cases, is the first event to point to disease of the kidneys. The anasarca may be great, or in other cases so slight as scarcely to attract the attention of the patient. Dropsy is more constant and marked in the large white kidney than in other forms of the chronic disease. In a certain proportion of cases it is wanting, and the great majority of these in the hard, contracted kidney. In this form the amount of albumen in the urine is generally small. The presence of casts in the sediment of urine is important. Epithelial casts, as has already been stated, are indicative of the acute affection; disintegrated epithelial casts, or granular casts, as they are termed, denote the chronic affection. Casts, in which the epithelial cells are filled with fat, are known as fatty casts, and are significant of an abnormal amount of fat in the secretory tubes. Hyaline or waxy casts, as already stated, have an important significance according to their size. Granular and large waxy casts, one-five hundredth of an inch in diameter, in abundance, indicate extensive destruction of the secretory tissue.

It is said that waxy casts may be dissolved by urine rendered alkaline by decomposition of urea. Spanæmia is generally marked, and is doubtless due, in part at least, to the loss of albumen. Pallor and œdema combined present an appearance highly characteristic of this disease, which contrasts strongly with the œdematous but turgid appearance of the face in certain cardiac lesions. The digestive system presents symptoms of importance; vomiting and purging are marked in many cases. It is probably an effort at vicarious elimination, and in this view, unless it persists to a degree which seriously interferes with alimentation, should be regarded as conservative. It may be mentioned here that the urea is converted, by contact with the mucus of the stomach, into carbonate of ammonia, which produces inflammation of the lining membrane of the gastro-intestinal canal, and in this way ultimately checks this mode of discharge of urea from the system. As in the acute affection, so here effusion into the serous cavities is liable to occur, and œdema of the lungs and glottis is an accident which may prove rapidly fatal.

The most important symptoms are those referable to the nervous system. They are cephalalgia, neuralgic pains, impaired vision, deafness, hemiplegia, paraplegia, paralysis, sometimes of a single member, coma and convulsions. Coma may precede or accompany the convulsions. In some cases it occurs without convulsions. It is generally developed gradually. The convulsions are epileptiform in character, and vary greatly in severity in different cases. Uræmia, as a rule, is quickly induced if the urea accumulates speedily, but if the accumulation be slow, the system acquires a tolerance of this, as of other toxic agents, and the effect may be postponed. It is of vast importance, so far as the prognosis is concerned, to discriminate between the acute and chronic affections. The objects of treatment in the former relate to subduing inflammation, removal of the dropsical effusion, and the elimination of urea, thus protecting the system from its poisonous effects until the integrity of the natural channels for its discharge is re-established.

In the chronic forms, involving as they do serious structural changes, which sooner or later prove fatal, unless life is destroyed by some intercurrent affection, the treatment can only be palliative, and serve to ward off the impending danger and defer the fatal result. As in the acute affection, the therapeutical indications refer to the removal of the dropsy and the elimination of urea.

Diuretics have been recommended in view of the fact that they, by increasing the secretion of urine, wash out the renal tubes, and remove mechanically the obstruction due to the accumulation of disintegrated epithelium and granular matter. Hydragogue cathartics and sudorific remedies meet the indications in these diseases, and are far more reliable than diuretics, which in the great majority of cases cannot be depended upon, as it is obvious that the organ upon which their specific action is exerted is more or less impaired, and its function, to a greater or less extent, suspended. In the administration of cathartics care, of course, should be exercised not to push them too far. Though this class of remedies, when judiciously administered, can frequently be borne by the patient without inconvenience for a considerable length of time. Astringents have been reccommended to restrain the excretion of albumen, but experience does not seem to accord to them much therapeutical efficacy. It is unnecessary to refer to the importance of improving the general health; of sustaining the patient by a nutritious diet and tonics, and counteracting the tendency to anæmia by preparations of iron.

